# Mannose Inhibits the Pentose Phosphate Pathway in Colorectal Cancer and Enhances Sensitivity to 5-Fluorouracil Therapy

**DOI:** 10.3390/cancers15082268

**Published:** 2023-04-13

**Authors:** Sadaf Al Hadeethi, Chirine El-Baba, Khaled Araji, Berthe Hayar, Israa Ahmad Cheikh, Riyad El-Khoury, Julnar Usta, Nadine Darwiche

**Affiliations:** 1 Department of Biochemistry and Molecular Genetics, American University of Beirut, Beirut 1107-2020, Lebanon; 2 Department of Pathology and Laboratory Medicine, American University of Beirut, Beirut 1107-2020, Lebanon

**Keywords:** colorectal cancer, pentose phosphate pathway, mannose, 5-fluorouracil, cancer therapeutics

## Abstract

**Simple Summary:**

5-fluorouracil (5-FU) has been the treatment of choice against colorectal cancer (CRC) for the past six decades. However, 5-FU exhibits high toxicity and drug resistance in CRC patients, highlighting the need for less toxic and more efficient treatments. The pentose phosphate pathway (PPP) is upregulated in cancer cells and promotes their survival. Recently, mannose has been reported to halt tumor growth and impair the PPP. We studied the effect of mannose, alone and in combination with 5-FU in human CRC cells and animal models. We have shown that mannose alone or in combination with 5-FU downregulated the PPP and enhanced the sensitivity of CRC cancer cells and tumors in mice to 5-FU. Therefore, this research may pave the way for better patient care.

**Abstract:**

Colorectal cancer (CRC) is one of the leading cancers and causes of death in patients. 5-fluorouracil (5-FU) is the therapy of choice for CRC, but it exhibits high toxicity and drug resistance. Tumorigenesis is characterized by a deregulated metabolism, which promotes cancer cell growth and survival. The pentose phosphate pathway (PPP) is required for the synthesis of ribonucleotides and the regulation of reactive oxygen species and is upregulated in CRC. Mannose was recently reported to halt tumor growth and impair the PPP. Mannose inhibitory effects on tumor growth are inversely related to the levels of phosphomannose isomerase (PMI). An in silico analysis showed low PMI levels in human CRC tissues. We, therefore, investigated the effect of mannose alone or in combination with 5-FU in human CRC cell lines with different p53 and 5-FU resistance statuses. Mannose resulted in a dose-dependent inhibition of cell growth and synergized with 5-FU treatment in all tested cancer cell lines. Mannose alone or in combination with 5-FU reduced the total dehydrogenase activity of key PPP enzymes, enhanced oxidative stress, and induced DNA damage in CRC cells. Importantly, single mannose or combination treatments with 5-FU were well tolerated and reduced tumor volumes in a mouse xenograft model. In summary, mannose alone or in combination with 5-FU may represent a novel therapeutic strategy in CRC.

## 1. Introduction

Colorectal cancer (CRC) is the third most commonly diagnosed cancer type and the second leading cause of cancer-related deaths [1,2]. The five-year survival rate declines from 90% to 70% in early CRC cases and in locally advanced CRC cases to reach 15% in metastatic CRC cases [3]. Regardless of the advancements made in systemic therapy, the survival rate for patients with metastatic CRC is still low. One of the main reasons for this low survival is related to the treatment’s failure, which is caused by innate or acquired drug resistance in 90% of patients [4].

5-Fluorouracil (5-FU) is a chemotherapeutic drug commonly used to treat different malignancies such as gastric, pancreatic, breast, ovarian, and head and neck cancers. Furthermore, 5-FU is the drug of choice for CRC treatment [5] and is present in the majority of CRC therapeutic regimens. However, 5-FU drug resistance is frequently encountered in patients; therefore, it is crucial to overcome this drug resistance [6].

The deregulation of cancer metabolism is an emerging hallmark of cancer [7]. There is a shift in many cancer cells from oxidative phosphorylation (OXPHOS) towards lactic acid fermentation, even in the presence of oxygen. This phenomenon is known as “aerobic glycolysis” or “Warburg effect” and may present a novel target for CRC therapy [8]. Although OXPHOS results in more ATP production per molecule of glucose, ATP formation is, however, faster via glycolysis. Additionally, upregulated glycolysis leads to increased glycolytic metabolites that feed different branching metabolic pathways, among which is the pentose phosphate pathway (PPP), facilitating the synthesis of macromolecules required to support the rapid proliferation of tumor cells [9].

The PPP, also known as the hexose monophosphate shunt or the phosphogluconate pathway, is a glucose metabolic pathway. The PPP is involved in ribose-5-phosphae (R5P) generation and preserves cytosolic reduction–oxidation (redox) homeostasis [10]. Unlike the TCA cycle and glycolysis, ATP is not produced by the PPP. However, it is a main source of NADPH and R5P, which are critical for cell survival and proliferation. NADPH is a reducing cofactor required for the biosynthesis of fatty acids, steroids, nucleotides, and non-essential amino acids, whereas R5P is required for the synthesis of nucleotides [11]. Thus, the PPP serves as a major glucose catabolic and reducing anabolic pathway fundamental for neoplastic transformation [10]. Moreover, the PPP enzymes, among which is the glucose-6-phosphate dehydrogenase (G6PD), are shown to be crucial for cancer prevention and treatment [10].

G6PD, the first and rate-limiting enzyme of the PPP oxidative phase, is upregulated in several cancer types. The overexpression of G6PD affects DNA synthesis, DNA repair, cell cycle regulation, redox homeostasis, proliferation, epithelial–mesenchymal transition, invasion, and metastasis, creating suitable and supportive conditions for the growth and survival of cancerous cells [12], while enhanced G6PD activity stimulates the resistance of tumor cells to chemotherapeutic drugs. Thus, G6PD inhibition was shown to be beneficial in reversing chemotherapy resistance in tumor cells [12].

D-Mannose (Mannose) is a natural C-2 epimer of glucose [13]. It possesses anti-bacterial, anti-inflammatory, and immune-regulatory activities, and it was recently shown to have anti-tumor activities [14]. Once mannose enters the cell via glucose transporters (GLUT1 and GLUT2) [13], it undergoes phosphorylation via hexokinase (HK) into mannose-6-phosphate (M6P) [15]. M6P is either isomerized by phosphomannose isomerase (PMI) to fructose-6-phosphate (F6P), involved in N-glycosylation by phosphomannose mutase 2 (PMM2), or catabolized by DN-9-phosphosynthase (KPS) for 2-keto-3-deoxy-D-glycero-galacto-nononic acid (KDN) synthesis [15]. The fate of M6P depends on the ratio of PMI to PMM2. High ratios induce M6P catabolism, while low ratios lead to the accumulation of M6P to interfere with glucose metabolism. Mannose has recently gained interest in research regarding its anti-cancer effects via glucose metabolism disturbance [10,16]. Gonzalez et al. have shown that mannose did not alter the intracellular levels of glucose; however, it was poorly metabolized within the cell and exerts effects on different metabolic pathways. The intracellular accumulation of mannose in the form of M6P leads to the suppression of three enzymes engaged in glucose metabolism, namely HK, phosphoglucose isomerase, and G6PD, therefore impairing glycolysis and the PPP [17]. The intracellular levels of PMI control the response to mannose. High PMI levels result in mannose insensitivity in cells while the depletion of PMI by RNA interference reverses sensitivity to mannose [17].

We studied whether inhibiting the PPP, using mannose alone or in combination with 5-FU, halts tumor progression and sensitizes CRC to conventional therapy. We used human CRC cell models with different p53 and 5-FU resistance statuses and a mouse model of xenografted CRC cells. We characterized the growth suppressive and cell death effects of mannose and 5-FU single treatments or in combination with 5-FU in CRC cells with different p53 and 5-FU sensitivity statuses and studied their anti-tumor potential in CRC using a xenograft mouse model. This study provides evidence for enhanced therapeutic strategies that target the metabolism in CRC using mannose alone or in combination with 5-FU.

## 2. Materials and Methods

### 2.1. In Silico Analysis of PMI mRNA Expression and Protein Levels in CRC

PMI mRNA expression and protein levels were determined in silico in colorectal tumors versus normal counterparts and other types of tumors. PMI mRNA expression levels were evaluated in colorectal adenocarcinoma, the rectum, and a normal colon. Collected data were obtained from The Human Protein ATLAS database, and boxplots were obtained from the Oncomine website (www.oncomine.com).

### 2.2. Cell Lines and Cell Culture Conditions

We have used the normal-like human colon epithelial cell line (NCM460D) and three isogenic human CRC cell lines: HCT116 wild-type for p53 (HCT116), p53 null (HCT116 p53^−/−^), and 5-FU-resistant HCT116 (HCT116-5FUR). The NCM460 cell line was purchased from INCELL Corporation, LLC San Antonio, TX, USA. The HCT116 cell line was obtained from the American Tissue Culture Collection, ATCC, Manassas, VA, USA. The HCT116 p53^−/−^ cells were provided by Carlos Maria Galmarini, PharmaMar, Madrid, Spain. The HCT116-5FUR cell line was generated in our laboratory from the parental HCT116 cells [18]. NCM460D cells were cultured in M3:Base media (INCELL) supplemented with 10% fetal bovine serum (FBS) (Sigma-Aldrich, St. Louis, MO, USA). HCT116 and HCT116-5FUR cells were cultured in an RPMI 1640 (Lonza, Basel, Switzerland) medium supplemented with 10% FBS (Sigma-Aldrich), 100 U/mL of penicillin-streptomycin antibiotics (Lonza), and 1 mM of sodium pyruvate solution (Lonza). HCT116 p53^−/−^ cells were cultured in a DMEM medium (Lonza) supplemented with 10% FBS, 100 U/mL of penicillin-streptomycin, 1 mM of sodium pyruvate, and 1× MEM non-essential amino acid (Sigma-Aldrich). All cells were incubated in a humidified incubator (95% air, 5% CO_2_) at 37 °C.

### 2.3. Drugs and Compounds

5-Fluorouracil (Sigma) was dissolved in dimethyl sulfoxide (DMSO) at a concentration of 10^−2^ mol/L and stored at −20 °C. D-mannose (Sigma) was dissolved in distilled water at a concentration of 1 M and stored at −20 °C.

### 2.4. cDNA Synthesis and Quantitative Real-Time Polymerase Chain Reaction (qRT-PCR)

RNA was extracted using a TRIzol reagent (Sigma-Aldrich) according to manufacturer’s instructions. RNA concentrations were measured using a DeNovix DS-11 spectrophotometer. Total RNA (1 μg) was reverse transcribed into cDNA using the iScript™ cDNA Synthesis Kit (Bio-Rad, Hercules, CA, USA), according to manufacturer’s instructions.

Primer sequences used to detect PMI transcripts (Macrogen, Seoul, Republic of Korea) are 5′-ATGTGCCAACCCTGTGTGAA-3′ for the forward sequence and 5′-TTCATGGCACACGAAGGACA-3′ for the reverse sequence. The sequences for β-actin (Macrogen) are 5′-CTCACCATGGATGATGATATCGC-3′ for the forward sequence and 5′-AGGAATCCTTCTGACCCATGC-3′ for the reverse sequence. Real-time PCR amplification reactions were performed using iTaq™ Universal SYBR^®^ Green Supermix (Bio-Rad), and real-time PCR was performed using a CFX96 Real-Time PCR machine (Bio-Rad). Analysis was performed using the 2^−∆CT^ method.

### 2.5. Thiazolyl Blue Tetrazolium Bromide Assay

The thiazolyl blue tetrazolium bromide dye (MTT, Sigma Aldrich) was used according to the manufacturer’s instructions. The absorbance was measured at 595 nm using an enzyme-linked immunosorbent assay (ELISA) microplate reader (Multiskan Ex).

### 2.6. Trypan Blue Cell Viability Assay

Cells were seeded in 24-well plates in triplicate and treated the next day with different mannose concentrations for up to 72 h. Media including floating dead cells were collected, and adherent living cells were detached by trypsin EDTA and added to the media. From a 500 µL cell suspension, 50 µL was mixed with 50 µL of trypan blue, and then live/dead cells were counted using a hemocytometer.

### 2.7. Sulforhodamine B Assay

Cells were fixed using 50% trichloroacetic acid solution, washed, and incubated with Sulforhodamine B (SRB) salt to be stained by it. Bound SRB was solubilized with 10 mM Tris base solution pH = 10.5. The plates were read at an absorbance of 595 nm using the ELISA Multiskan Ex microplate reader.

### 2.8. Total Dehydrogenase Activity

Total proteins were extracted with a 1x lysis buffer (10 mM Tris-HCl pH 7.4, 150 mM NaCl, 15% glycerol, 1% Triton-X-100, 1 mM orthovanadate, 10 μg/mL leupeptin, and 1 mM PMSF) and quantified using the Bradford Assay (Bio-Rad, Hercules, CA, USA). A total of 50 μg of proteins from each sample were mixed with 50 μM of G6P, 75 μM of NADP+, and an assay buffer (50 mM Tris, 1 mM MgCl_2_, pH 8.6) for the assay. The total dehydrogenase basal activity of G6PD and 6PGD was determined spectrophotometrically by monitoring the change in absorbance at 340 nm for 10 min using the Tristar machine.

### 2.9. Nitroblue Tetrazolium Reduction Assay

Cells were seeded in 96-well plates in triplicate. Following media removal, 100 μL of nitroblue tetrazolium (NBT) (MERCK, Rahway, NJ, USA) (1 mg/mL) was added to each well and incubated for 1 h at 37 °C in a humidified 5% CO_2_ incubator. The cells were then washed with 100 μL of methanol per well. Formazan crystals obtained by NBT salt reduction were then solubilized by the addition of 120 μL potassium hydroxide (KOH) (2 M) and 140 μL DMSO. The reduced NBT dye was assessed by measuring the OD at 630 nm using an ELISA micro-plate reader. The percentage of reduced NBT was obtained from the ratio of absorbance obtained in treated cells to that of the control multiplied by 100. ROS levels were determined by subtracting the % NBT reduced from 100.

### 2.10. Cell Cycle Analysis

Cell cycle analysis was performed using the propidium iodide (PI) assay. Cellular pellets were incubated with 100 μL RNase (Roche Diagnostics, Basel, Switzerland) for 1 h, resuspended in up to 500 μL 1× PBS, stained with 30 μL PI (Sigma-Aldrich), and then incubated for 10 min in the dark. In total, 10,000 cells were collected and analyzed using a FACScan flow cytometer (Becton Dickinson, Franklin Lakes, NJ, USA), and cell cycle distribution was verified using BD FACSDIVA software.

### 2.11. Terminal Deoxynucleotidyl Transferase dUTP Nick-End Labeling (TUNEL) Assay

Cells were treated with mannose (50 mM), 5-FU (5 μM), mannose (50 mM) + 5-FU (5 μM), 5 μM ST1926 for the positive controls [18], and negative controls up to 72 h. Control and treated cell DNA fragmentation was detected by terminal deoxynucleotidyl transferase (TdT)-mediated dUTP nick-end labeling (TUNEL assay; Roche Diagnostics) according to the manufacturer’s recommendations. The incorporation of fluorescein-conjugated deoxy-UTP into nucleotide polymers was detected and quantified using flow cytometry (BD FACSAria). In total, 10,000 cells were acquired and analyzed using FACSDiva software (Becton-Dickinson, San Jose, CA, USA).

### 2.12. Western Blotting

Cells were treated for up to 3 days for short-term conditions and 6 days for long-term conditions with treatment replenishment on day 4. Total protein lysates were extracted using a Nonidet TMP 40-based lysis buffer (NP-40) and quantified using the Bio-Rad Bradford protein assay. Protein samples were separated via SDS-PAGE (8–12%) in reducing conditions and transferred to nitrocellulose membranes. The latter were blocked in 5% non-fat milk for 1 h and then incubated with primary antibodies against the following antigens at 4 °C overnight: G6PD (Abcam, Waltham, MA, USA) (1:1000), TKT (Cell Signaling, Danvers, MA, USA) (1:1000), Poly (ADP-ribose) polymerase (PARP) (Santa Cruz, Dallas, TX, USA) (1:1000), phosphorylated H2A histone family member X (γH2AX) (Cell Signaling) (1:1000), and GAPDH (Abnova, Taipei, Taiwan) (1:20,000). Membranes were incubated the next day for 1 h against the corresponding secondary antibodies at different optimized dilutions. The immunoreactive bands were visualized using a Clarity^TM^ western ECL substrate (ECL, Bio-Rad) and by the Chemidoc^TM^ MP imaging System (Bio-Rad). Densitometric ratios were calculated using ImageJ software for image processing and analysis in Java.

### 2.13. Mouse Xenograft Studies

The animal studies were approved and conducted according to institutional guidelines and approved by the Institutional Animal Care and Use Committee of the American University of Beirut (IACUC approval). The nonobese diabetic/severe combined immunodeficiency (NOD/SCID) mouse model was selected in our studies. We used a total number of 50 mice (24 males and 26 females) at 4 to 6 week old, and they were injected with 5 × 10^6^ HCT116 cells. The animals were randomly divided into 4 groups (Table 1). The control group received a vehicle (water), while the experimental groups were treated three times per week with either 10 mg/Kg body weight of 5-FU alone given intraperitoneally or with 20% mannose alone administered by gavage in addition to having 20% mannose in freely available drinking water or a combination of both treatments. The mice’s body weights and tumor volumes were measured twice per week and calculated using calipers and the formula: Tumor Volume = Length × (Width)^2^/2. Any signs of toxicity such as pain, distress, ill appearance, fur changes, and rhinitis were monitored. The endpoint of the experiment was 28 days or when weight loss reached 20–25% of the body weight.

### 2.14. Statistical Analysis

Results represent the average of three independent experiments ± the standard error of the mean (SEM) unless stated otherwise. Statistical significance is reported by two-way ANOVA post hoc Bonferroni’s multiple comparisons using GraphPad Prism version 9. *p* values less than 0.05 were considered significant from the control. *, **, and *** indicate *p* values less than 0.05, 0.01, and 0.001, respectively.

## 3. Results

### 3.1. PMI Levels Are Low in Human Colorectal Cancer Tissues

**Figure 1:**
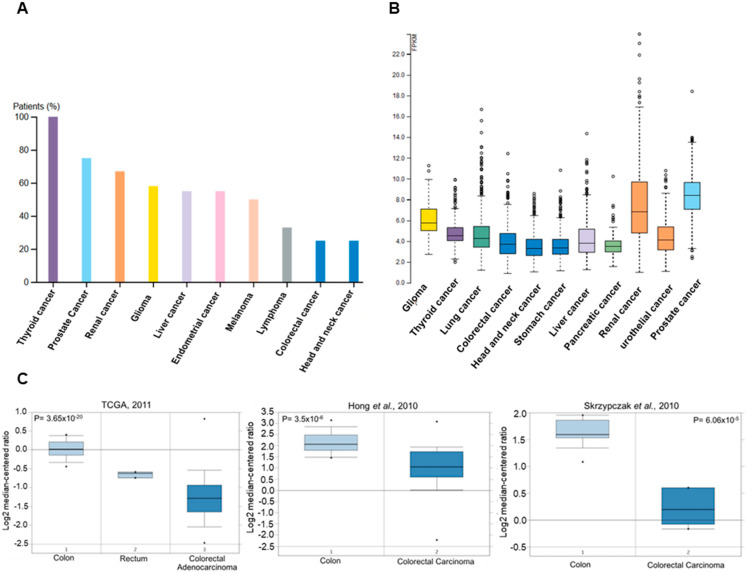
PMI levels are attenuated in CRC tissues. (**A**) PMI levels in the cytosol were assessed using anti-PMI antibodies in patients with different types of tumors. (**B**) RNA-seq analysis for PMI was performed on different types of tumors. Data were obtained from ATLAS protein expression (data extracted on Nov 2019). (**C**) Expression levels of PMI mRNA [19,20].

Mannose sensitivity is dependent on the levels of PMI. Cells with low levels of PMI are sensitive to mannose, whereas those with high levels of PMI are resistant due to mannose conversion into glucose. Thus, we conducted an in silico analysis of PMI levels in publicly available databases to determine the PMI expression and protein levels in human tissues (Figure 1). Low levels of PMI proteins in CRC patients were detected compared to other types of tumors (Figure 1A). Moreover, CRC tissues showed lower PMI mRNA expression when compared to other types of tumors (Figure 1B). Human CRC tissues showed lower PMI mRNA expression compared to adjacent normal counterparts (Figure 1C) [19,20]. Then, we measured the PMI transcript levels in the CRC cell lines (HCT116, HCT116 p53^−/−^, and HCT116-5FUR) and compared them to the normal-like colon cells (NCM460D). The CRC cells did not show significant differences in PMI expression compared to the normal-like colon cells (Figure S1).

### 3.2. Mannose Inhibits the Growth of Human CRC Cells with Different p53 and 5-FU Resistance Statuses

**Figure 2:**
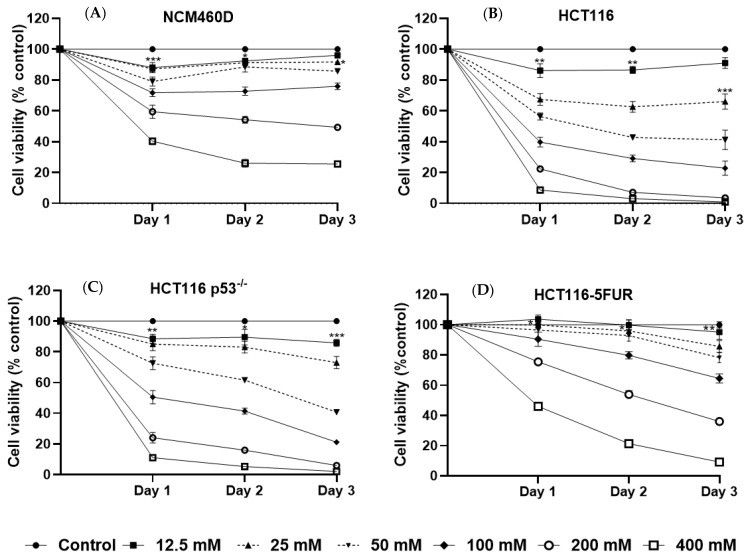
Mannose reduces cancer cell viability. Effect of mannose on (**A**) normal-like colon cells NCM460D, (**B**) HCT116, (**C**) HCT116 p53^−/−^, and (**D**) HCT116-5FUR cells. Results are expressed as a percentage of the control and represent the average of at least three independent experiments ± SEM. The asterisk represents the lowest concentration showing a significant decrease in cell viability in treated versus control cells. Significance from the control is indicated by * *p* < 0.05, ** *p* < 0.01, and *** *p* < 0.001.

Low PMI levels in CRC suggest high mannose sensitivity. Therefore, we investigated the growth-suppressive effects of mannose in CRC cells and normal-like colon cells. HCT116, HCT116 p53^−/−^, HCT116-5FUR, and NCM460D cells were treated for up to 72 h with different concentrations of mannose. Mannose significantly reduced the growth of tested CRC cell lines in a dose- and time-dependent manner (Figure 2B–D). However, mannose up to 100 mM had minimal effects on the viability of NCM460D cells (Figure 2A). The MTT results were confirmed by a trypan blue dye exclusion assay where the same trends with respect to cell viability were observed (Figure S2) without significant effects on cell death (Figure S3). To verify the stability of the mannose treatment, we conducted an MTT assay to confirm the need to replenish mannose treatment daily. Mannose-treated HCT116 cells with or without daily replenishment presented similar growth inhibition rates, thus confirming the non-requirement of daily mannose replenishment (Figure S4).

### 3.3. Mannose Synergizes with 5-FU in Reducing Colorectal Cancer Cell Viability

**Figure 3:**
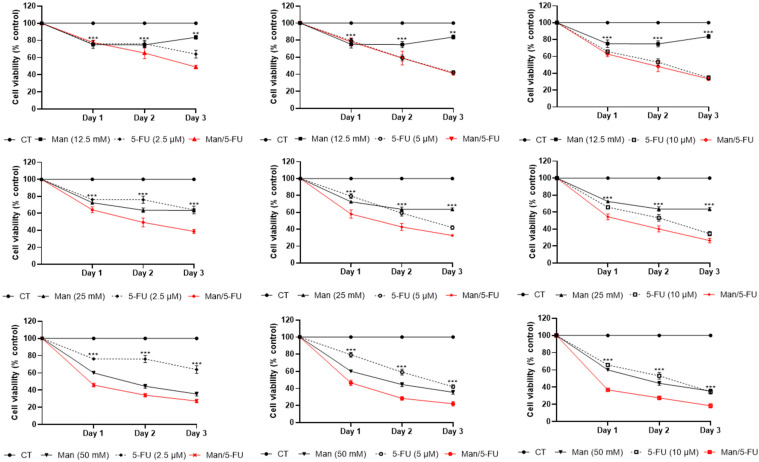
Mannose and 5-FU combination treatments display synergistic interactions in HCT116 cells. Results are expressed as a percentage of control and represent the average of three independent experiments ± SEM. The asterisk represents the lowest concentration showing a significant decrease in cell viability in treated versus control cells. Significance from the control is indicated by ** *p* < 0.01, and *** *p* < 0.001.

**Figure 4:**
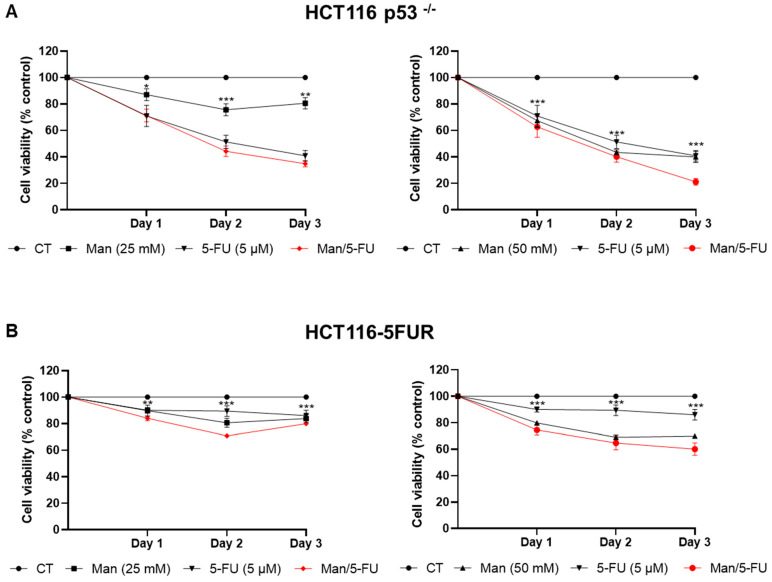
Mannose and 5-FU combination treatments sensitize the human colorectal cancer cells relative to the 5-FU treatment. (**A**) HCT116 p53^−/−^; (**B**) HCT116-5FUR cells. MTT results are expressed as a percentage of the control and represent the average of three independent experiments ± SEM. The asterisk represents the lowest concentration showing a significant decrease in cell viability in treated versus control cells. Significance from the control is indicated by * *p* < 0.05, ** *p* < 0.01, and *** *p* < 0.001.

Combination treatments of mannose and 5-FU reduced the viability of HCT116 cells at all tested concentrations (Figure 3). To further confirm the synergistic effect and identify the best combination of mannose and 5-FU with the lowest possible cytotoxicity, we analyzed the results obtained by MTT using the computational Compusyn software. Synergy was observed in several conducted combinations where CI < 1 (red) (Table 2). Biologically significant synergy (CI = 0.33 and CI = 0.48) was detected at 24 h when 25 mM or 50 mM mannose was combined with 5 µM 5-FU (Table 2). To validate the results of the MTT assay and to verify the synergistic effect of the selected combination treatments in HCT116 cells, we used the SRB cell density assay. HCT116 cells were treated with mannose alone or in combination with 5-FU for up to 72 h. Both assays revealed similar growth inhibition trends, thus confirming MTT results (Figure S5). The synergistic effect of the combination treatment was also observed in HCT116 p53^−/−^ and HCT116-5FUR cells (Figure 4). While for the 5-FU single treatment, we observed that HCT116-5FUR cells were relatively more resistant to the drug than the parental HCT116 and HCT116 p53^−/−^ cells. In fact, 5 µM 5-FU treatments for 3 days resulted in a 58%, 60%, and 14% decrease in viability with respect to HCT116, HCT116 p53^−/−^, and HCT116-5FUR cells, respectively (Figure 3, Figure 4).

### 3.4. Mannose Alone or in Combination with 5-FU Reduces the Total Dehydrogenase Activity of Key PPP Enzymes in CRC Cells

**Figure 5:**
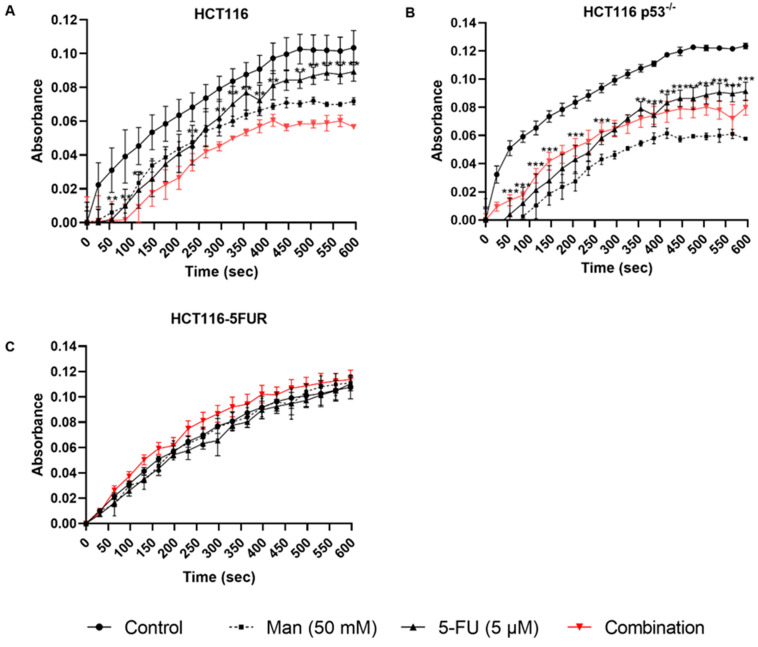
Effect of mannose alone or in combination with 5-FU on the total dehydrogenase activity of the PPP in human colorectal cancer cells. (**A**) HCT116; (**B**) HCT116 p53^−/−^; (**C**) HCT116-5FUR cells. Total dehydrogenase activity was determined in triplicate measurements and was measured spectrophotometrically by monitoring the change in absorbance at 340 nm for 5 min. Results represent an average of three independent experiments ± SEM. ** *p* < 0.01, and *** *p* < 0.001 are considered statistically significant.

To explore the effect of mannose on the oxidative and non-oxidative phases of the PPP, G6PD and TKT protein levels were determined in HCT116- and HCT116 p53^−/−^-treated cells with mannose and/or 5-FU. None of the tested conditions modified G6PD nor TKT protein levels (Figures S6 and S7). Afterwards, the total dehydrogenase basal activity of the oxidative phase of the PPP (G6PD and 6PGD) was evaluated in the CRC cells HCT116, HCT116 p53^−/−^, and HCT 5FUR (Figure 5). Cells were treated with mannose alone or/and 5-FU for 48 h. Mannose alone or in combination with 5-FU decreased the total dehydrogenase activity in HCT116 and HCT116 p53^−/−^ cells, where mannose showed a 50% reduction in total dehydrogenase activity compared to the control (Figure 5A,B). However, no tested treatment significantly affected the total dehydrogenase activity in HCT116-5FUR cells (Figure 5C). In summary, mannose alone or in combination with 5-FU reduced the activity of total dehydrogenase of key enzymes of the PPP in CRC cells with different p53 statuses but not in 5-FU-resistant cells.

### 3.5. Mannose Alone or in Combination with 5-FU Induces ROS Production in Colorectal Cancer Cells

**Figure 6:**
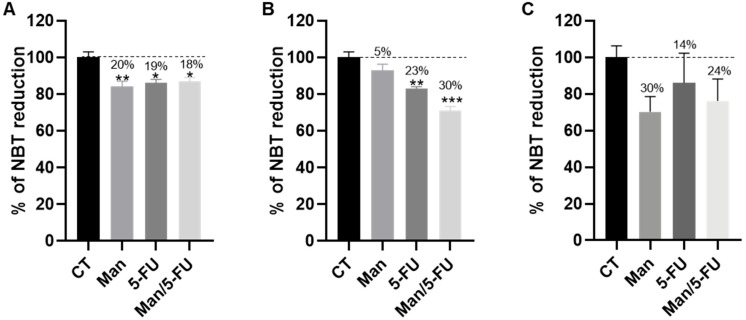
Effect of mannose and 5-FU single and combination treatments on reactive oxygen species (ROS) production in human CRC cells. (**A**) HCT116, (**B**) HCT116 p53^−/−^, and (**C**) HCT116-5FUR cells were treated with mannose (man 50 mM) and/or 5-FU (5 µM) for 24 h. ROS levels represent an average of three independent experiments ± SEM. * *p* < 0.05, ** *p* < 0.01, and *** *p* < 0.001 are considered statistically significant.

To further study the mechanism of action of mannose in combination with 5-FU, we investigated ROS production in CRC cells. HCT116, HCT116 p53^−/−^, and HCT116-5FUR cells were treated with mannose alone or in combination with 5-FU for up to 24 h. ROS production was assessed by an NBT reduction assay. HCT116 and HCT116 p53^−/−^ cells treated with the combination of mannose/5-FU for one day showed a significant decline in the reduced NBT by 20% and 30%, respectively (Figure 6). Thus, mannose alone or in combination with 5-FU for 24 h induces oxidative stress in CRC cells that are not 5-FU-resistant.

### 3.6. Mannose in Combination with 5-FU Causes S-Phase Cell Cycle Arrest and Sub-G_1_ Accumulation in Colorectal Cancer Cells

**Figure 7:**
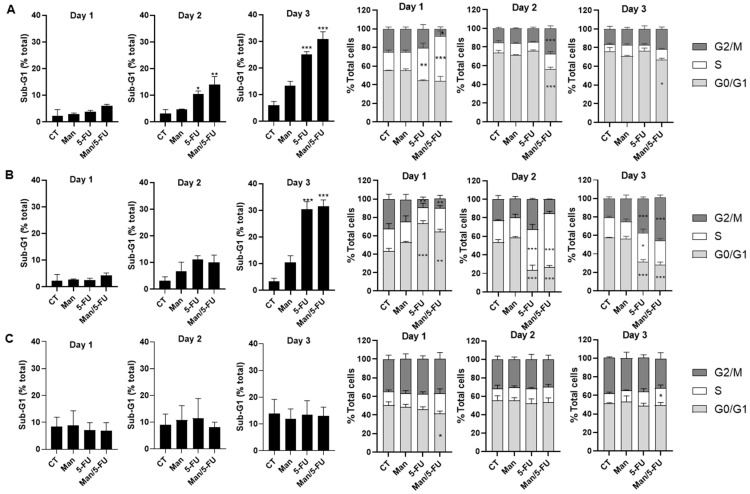
Mannose and 5-FU treatments induce S-phase arrest and increase sub-G1 cell accumulation in colorectal cancer cells. Cells were treated with mannose (50 mM) and/or 5-FU (5 µM) for up to 3 days. (**A**) HCT116, (**B**) HCT116 p53^−/−^, and (**C**) HCT116-5FUR cells. The sum of G0/G1, S, and G2/M phases is a percentage of non-dead cells. Percentage cells in the G0/G1 phase are calculated as 100 − (S + G2/M). Results represent the average of three independent experiments (±SEM). Significance from the control is indicated by * *p* < 0.05, ** *p* < 0.01, and *** *p* < 0.001.

We investigated the mechanism of cell growth inhibition induced by the combination treatment via cell cycle analysis. HCT116, HCT116 p53^−/−^, and HCT116-5FUR cells were treated with mannose (50 mM) and 5-FU (5 μM) alone or in combination for up to 72 h. Cells treated with mannose/5-FU showed a significant cell accumulation in the sub-G_1_ region in a time-dependent manner in HCT116 and HCT116 p53^−/−^ but not in HCT116-5FUR cells (Figure 7). The S-phase arrest was observed upon the combination treatment on day 1, day 2, or day 3 in HCT116, HCT116 p53^−/−^, and HCT116-5FUR cells, respectively. The accumulation of mannose- and 5-FU-treated HCT116 and HCT116 p53^−/−^ cells in the sub-G_1_ phase is presumably apoptotic.

### 3.7. 5-FU Alone or in Combination with Mannose Induces DNA Damage in Colorectal Cancer Cells

**Figure 8:**
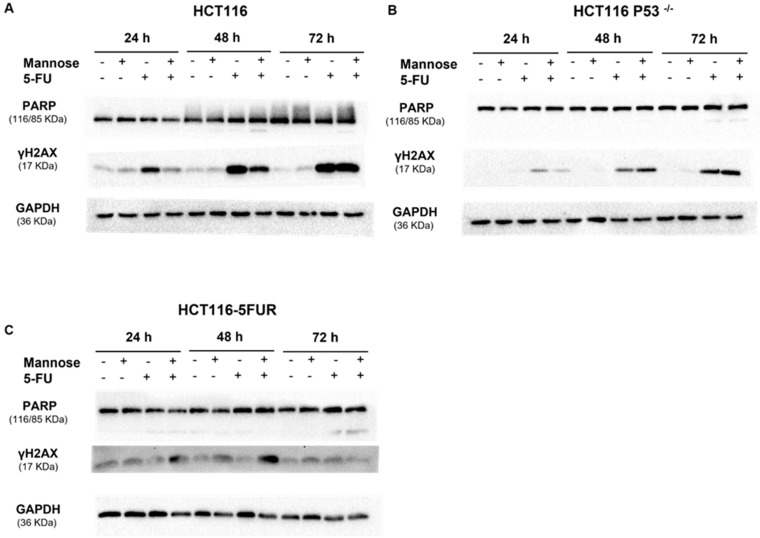
Mannose- and 5-FU-treated colorectal cancer cells induce DNA damage. (**A**) HCT116, (**B**) HCT116 p53^−/−^ and (**C**) HCT116 5FU resistant (HCT116-5FUR) cells were treated with mannose (50 mM) and/or 5-FU (5 µM) for up to 72 h. Total SDS protein lysates were immunoblotted against PARP and γ H2AX antibodies. GAPDH was used as a loading control. This is a representative blot of three independent experiments.

Since CRC cells accumulate in the presumably apoptotic sub-G1 phase upon a combination treatment of mannose and 5-FU, we further investigated the mechanism of cell death. PARP cleavage by caspases is considered a hallmark of apoptosis, while γH2AX is a DNA damage indicator. A minor PARP cleavage was detected in the different tested CRC cell lines after 3 days of mannose/5-FU combination treatments (Figure 8). 5-FU single treatment or in combination with mannose resulted in γH2AX-increased protein levels in HCT116, HCT116 p53^−/−^, and HCT116-5FUR cells (Figure 8). We then studied the effects of the different treatments on the most sensitive HCT116 cells. No major PARP cleavage was detectable up to day six (Figure S8). HCT116-treated cells with the 5-FU or 5-FU/mannose combination showed more pronounced γH2AX protein accumulation at day six (Figure S8). Our results indicate that 5-FU and single mannose or combination treatments cause DNA damage in CRC cells with different p53 statuses and 5-FU sensitivity. We conducted the TUNEL assay to further verify whether the cell death mechanism induced upon treatment with mannose alone or in combination with 5-FU is due to apoptosis. HCT116 cells were treated with mannose (50 mM) alone or in combination with 5-FU (5 µM). We did not observe any TUNEL positivity upon three days of treatment with respect to HCT116 cells (Figure S9).

### 3.8. Mannose Alone or in Combination with 5-FU Reduces Tumor Growth in Xenografted Mice

**Figure 9:**
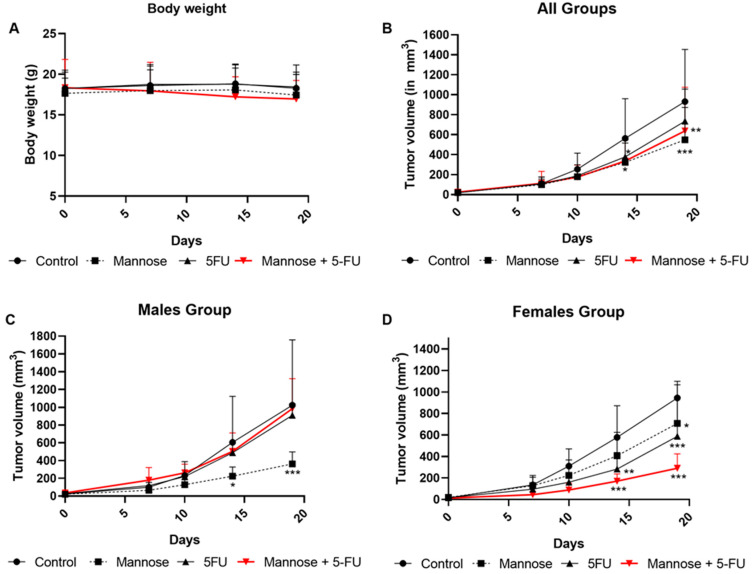
Body weights and tumor volumes of NOD/SCID mice treated with mannose alone, 5-FU, or in combination. Here, 5 × 10^6^ HCT116 cells were injected subcutaneously into the flank of NOD/SCID mice. After tumor development, mice were divided into 4 groups: control, mannose, 5-FU, and mannose/5-FU. (**A**) Body weights were measured over time and represented among the four groups. Tumor volumes were measured over time and represented among the four groups, including (**B**) males and females, (**C**) males only, and (**D**) females only. * *p* < 0.05, ** *p* < 0.01, and *** *p* < 0.001 are considered statistically significant.

We investigated the anti-tumor potential of mannose alone or in combination with 5-FU using a CRC xenograft mouse model. A total of 5 × 10^6^ HCT116 cells were injected subcutaneously into the flank of immunocompromised NOD/SCID mice. We studied the effect of single and combination treatments using mannose with or without 5-FU in xenografted tumor cells. Mannose was freely available in drinking water at 20% and by oral gavage three times per week (200 µL of 20% solution) as these concentrations are demonstrated to be nontoxic and well tolerated in mice [17]. 5-FU (10 mg/kg BW) is non-toxic to mice [21] and was administered intraperitoneally three times per week in parallel with the mannose treatment. Mice with palpable tumors around day seven were randomly divided into four groups. The control group received the vehicle alone while the experimental groups received mannose, 5-FU, and a combination of mannose and 5-FU. The different treatments were well tolerated in mice as no significant change in body weights was observed among the different groups (Figure 9A). We noticed a significant decrease in tumor volumes in mice treated with mannose alone or in combination with 5-FU compared to the control group (males and females combined) (Figure 9B). Unexpectedly, the combination treatment of mannose/5-FU did not reduce tumor volumes in treated male mice. Interestingly, male mice showed the most pronounced tumor growth inhibition in the mannose-treated group (Figure 9C), while female mice exhibited a less significant tumor volume reduction (Figure 9D). Female mice showed the most significant tumor volume reduction in the mannose/5-FU combination group (Figure 9D).

## 4. Discussion

CRC is the third most commonly diagnosed malignancy and the second leading cause of cancer-related deaths worldwide [1]. 5-FU-based chemotherapy is the first-line treatment for patients with advanced CRC [22]. Unfortunately, the five-year survival rate of metastatic CRC is still only 10–15% success [23]. Glycolysis and PPP-related enzymes are elevated in colorectal carcinoma specimens [24]. Thus, targeting the PPP is crucial for CRC treatment where many of its enzymes have shown to be potential targets in cancer therapy, among which is the G6PD [10]. G6PD is the rate-limiting enzyme of the PPP. G6PD is upregulated in several cancer types where it affects several cellular functions, creating a suitable and supportive environment for cancerous cell growth and survival. In addition, G6PD-enhanced activity stimulates resistance to chemotherapeutic drugs in cancer cells. Therefore, G6PD inhibition was shown to be beneficial in reversing chemotherapy resistance in malignant cells [12].

Mannose has recently gained interest regarding its anti-cancer effects via glucose metabolism disturbance [17]. We hypothesized that inhibiting the PPP using mannose alone or in combination with 5-FU in CRC may provide new therapeutic opportunities for CRC. Therefore, we aimed to inhibit the PPP by using mannose alone or in combination with 5-FU where combination treatments may enhance 5-FU efficacy. Sensitivity to mannose depends on intracellular PMI levels [17]. The in silico analysis of PMI levels in CRC specimens revealed that PMI mRNA and protein levels are low in CRC compared to other cancer types or to normal colonic counterparts, which explains their sensitivity to mannose. However, no significant difference in PMI expression was observed in our tested CRC cell lines compared to normal-like colon cells. Recent studies have shown the growth-suppressive effect of mannose in lung cancer [25], glioma [26], osteosarcoma, and pancreatic cancer cell lines [17]. In the present study, we have demonstrated that mannose resulted in a time- and dose-dependent cell growth inhibition in HCT116, HCT116 p53^−/−^, and HCT116-5FUR cells at concentrations that do not affect the normal-like colon cells.

Mannose has shown synergistic effects with several chemotherapeutic drugs such as cisplatin and doxorubicin in osteosarcoma [17]; temozolomide and chemoradiotherapy in glioma [26,27]; and carboplatin in lung cancer cell lines [25]. Here, we found that mannose synergizes with 5-FU in CRC. The synergistic effect of the selected combination treatments was also observed on other CRC cell lines harboring different p53 and 5-FU resistance statuses. We observed S-phase cell cycle arrest but no apoptosis in CRC cells treated with the combination of mannose and 5-FU. The mannose/5-FU combination treatment was shown to induce G0/G1 cell cycle arrest in lung cancer cells [28]. 5-FU alone or in combination with mannose resulted in DNA damage in the different CRC cell lines with different p53 and 5-FU resistance statuses and increased ROS production only in 5-FU-sensitive cells. It remains to be determined whether the cell cycle arrest and cell death mechanisms induced by the different treatments are ROS-dependent. Mannose accumulation in the form of M6P led to glycolysis disruption by inhibiting three enzymes, among which is G6PD [17]. Mannose alone or in combination with 5-FU decreased the total dehydrogenase activity in HCT116 despite their different p53 status but not in the 5-FU-resistant cells.

In addition to in vitro studies, we aimed at assessing the impact of mannose alone or in combination with 5-FU in vivo. We xenografted HCT116 cells in NOD/SCID mice in order to study the anti-tumorigenic effects of mannose alone or in combination with 5-FU. The different treatments were well tolerated by all animals. We observed a significant decrease in tumor volumes in mice groups treated with mannose alone or in combination with 5-FU compared to the control group. Gender affects susceptibility to many diseases as well as the reaction to various drugs. It was shown that mannose serum levels in males were higher than that of females [29]. Interestingly, male mice showed the most pronounced tumor volume reduction in the mannose-treated group, while female mice exhibited a less significant tumor volume reduction. Chemotherapy has been used independent of sex; meanwhile, accumulating evidence shed light on a sex-related response to chemotherapeutic drugs, among which is 5-FU. Females usually experience higher toxicity towards 5-FU. Low drug clearance and decreased activity of dihydropyrimidine dehydrogenase that breaks down 5-FU, in females, affect drug efficacy and toxicity [30]. We have preliminary results that should be further validated, indicating that sex-related disparities may explain why female mice show the most significant tumor volume reduction in the 5-FU single treatment and in combination with mannose, while tumors in male mice were not affected by the combination treatment. On the other hand, a study showed that male mice were more sensitive to the 5-FU treatment than female mice [31]. However, the authors used a different mouse strain (BALB/c nude mice) where tumors grew more in male than in female animals, and mice were administered a three times higher dose of 5-FU [31]. Thus, the mannose anti-tumor effect in CRC may be affected by sex, which remains to be investigated in future studies.

## 5. Conclusions

We have shown that mannose alone or in combination with 5-FU reduces the activity of key enzymes in the PPP and sensitizes the treatment of CRC cells to 5-FU. Importantly, treatments with mannose alone or in combination with 5-FU are well tolerated in mice and result in tumor growth reduction. We noticed sex disparities in the male versus female mice treatment response, which need to be further investigated.

## Figures and Tables

**Table 1:** In vivo experimental groups distribution. F: Females; M: males.

Treatment	Control	Mannose	5-FU	Mannose/5-FU
Number of mice	12 (6 M, 6 F)	13 (6 M, 7 F)	13 (6 M, 7 F)	12 (6 M, 6 F)

**Table 2:** Mannose and 5-FU Compusyn study on HCT116-treated cells. Combination index (CI) values of CI = 1 additive effect, CI < 1 synergistic, and CI > 1 antagonistic. Red values indicate synergistic interactions.

24 Hours			48 Hours			72 Hours		
Mannose (mM)	5-FU (µM)	CI	Mannose (mM)	5-FU (µM)	CI	Mannose (mM)	5-FU (µM)	CI
12.5	2.5	2.11715	12.5	2.5	1.12495	12.5	2.5	0.89611
12.5	5	3.04617	12.5	5	1.22017	12.5	5	1.03834
12.5	10	0.73937	12.5	10	1.13527	12.5	10	1.24165
25	2.5	0.72892	25	2.5	0.79519	25	2.5	0.88039
25	5	0.48905	25	5	0.76761	25	5	0.96374
25	10	0.42279	25	10	0.94199	25	10	1.10926
50	2.5	0.29071	50	2.5	0.68224	50	2.5	0.94712
50	5	0.33398	50	5	0.56759	50	5	0.91218
50	10	0.15323	50	10	0.70285	50	10	0.95026

## Data Availability

All data generated or analyzed in this manuscript are included in this article and in Supplementary Files.

## Supplementary Materials

The following supporting information can be downloaded at: https://www.mdpi.com/article/10.3390/cancers15082268/s1, Figure S1: RT-qPCR analysis of PMI basal RNA levels., Figure S2: Effect of mannose treatment on the viability of normal and colorectal cancer cell lines., Figure S3: Effect of mannose treatment on the viable and dead cell counts in the normal-like colon and colorectal cancer cell lines., Figure S4: Effect of mannose replenishment on colorectal cancer HCT116 cell viability., Figure S5. Effects of mannose and 5-FU single and combination treatments on colorectal cancer cells., Figure S6: Mannose and/or 5-FU treatment did not alter G6PD nor transketolase protein levels., Figure S7: Mannose and/or 5-FU treatment did not alter G6PD nor transketolase protein levels., Figure S8: Mannose and 5-FU treatments of HCT116 cells induced DNA damage but no PARP cleavage., Figure S9: Mannose alone or in combination with 5-FU did not induce apoptosis in HCT116 cells.
