# Supplementary material for: Mannose Inhibits the Pentose Phosphate Pathway in Colorectal Cancer and Enhances Sensitivity to 5-Fluorouracil Therapy
Source: Cancers (Basel). 2023 Apr 13;15(8):2268. doi: 10.3390/cancers15082268 (PMC10137209; doi:10.3390/cancers15082268)
Supplement: Supplementary file 1 [file cancers-15-02268-s001.zip › cancers-2211780-supplementary.pptx]

## Slide 1
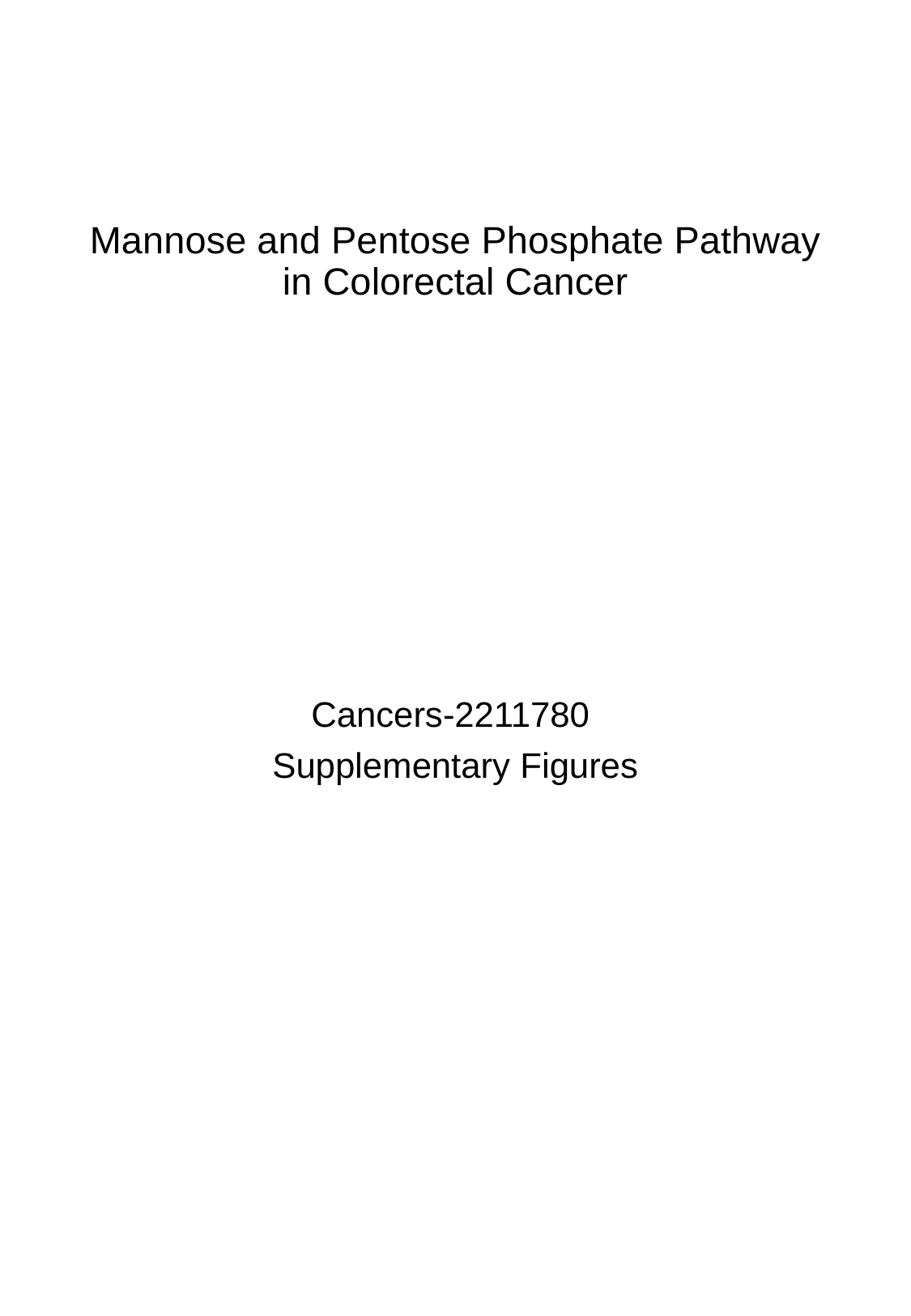

Mannose and Pentose Phosphate Pathway in Colorectal Cancer
Cancers-2211780
Supplementary Figures

## Slide 2
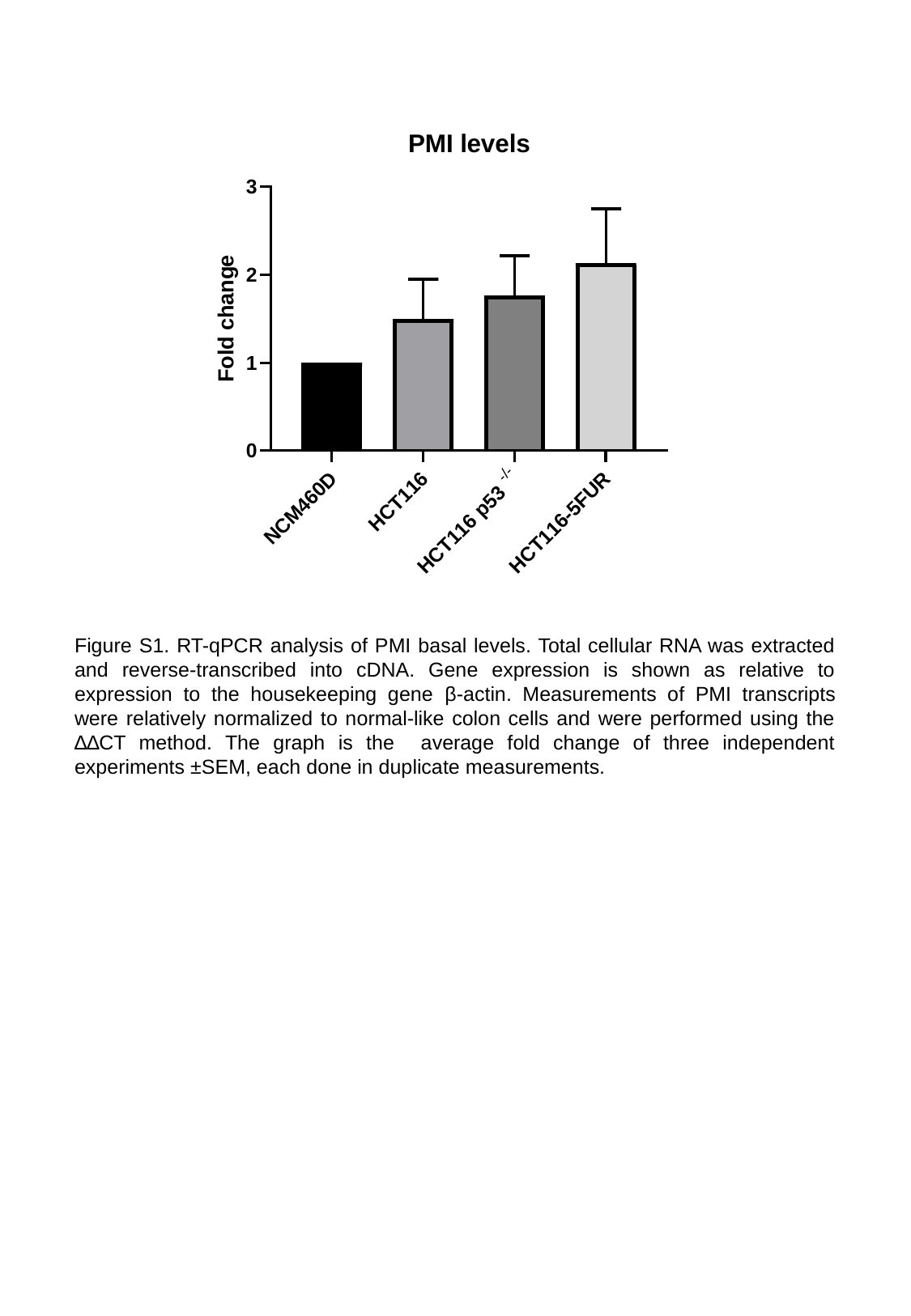

Figure S1. RT-qPCR analysis of PMI basal levels. Total cellular RNA was extracted and reverse-transcribed into cDNA. Gene expression is shown as relative to expression to the housekeeping gene β-actin. Measurements of PMI transcripts were relatively normalized to normal-like colon cells and were performed using the ∆∆CT method. The graph is the average fold change of three independent experiments ±SEM, each done in duplicate measurements.

## Slide 3
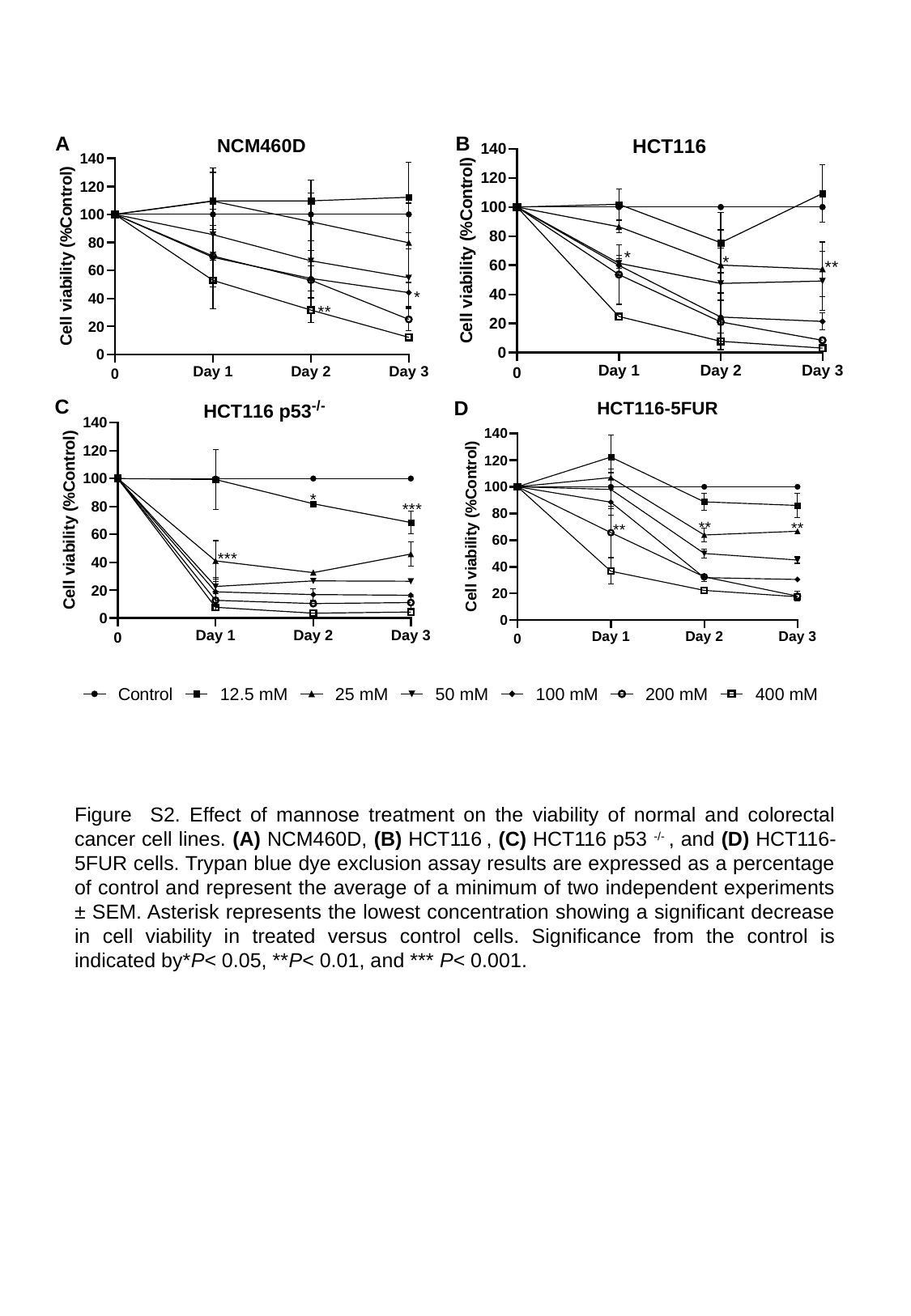

B
A
C
D
Figure S2. Effect of mannose treatment on the viability of normal and colorectal cancer cell lines. (A) NCM460D, (B) HCT116 , (C) HCT116 p53 -/- , and (D) HCT116-5FUR cells. Trypan blue dye exclusion assay results are expressed as a percentage of control and represent the average of a minimum of two independent experiments ± SEM. Asterisk represents the lowest concentration showing a significant decrease in cell viability in treated versus control cells. Significance from the control is indicated by*P< 0.05, **P< 0.01, and *** P< 0.001.

## Slide 4
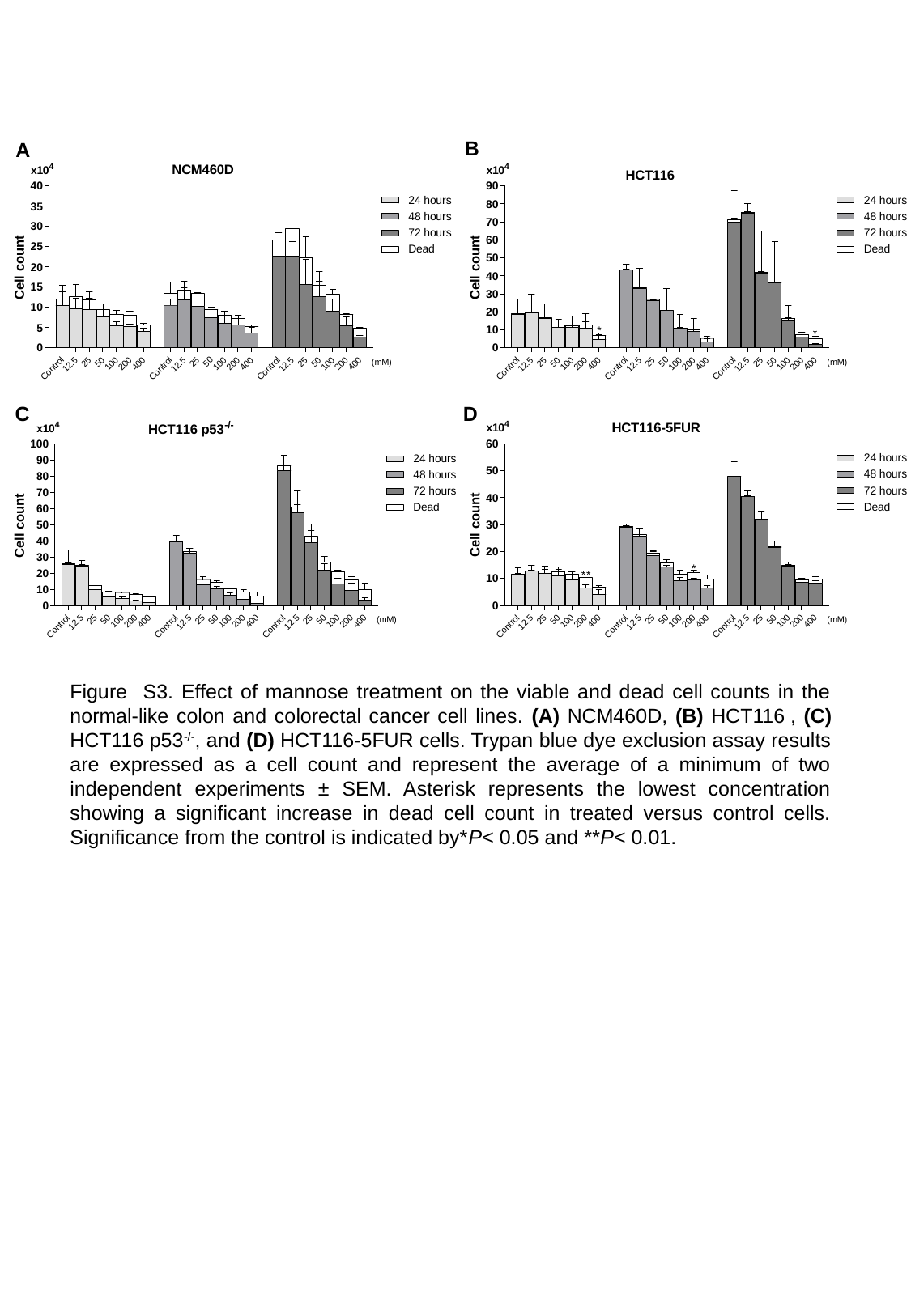

B
A
C
D
Figure S3. Effect of mannose treatment on the viable and dead cell counts in the normal-like colon and colorectal cancer cell lines. (A) NCM460D, (B) HCT116 , (C) HCT116 p53-/-, and (D) HCT116-5FUR cells. Trypan blue dye exclusion assay results are expressed as a cell count and represent the average of a minimum of two independent experiments ± SEM. Asterisk represents the lowest concentration showing a significant increase in dead cell count in treated versus control cells. Significance from the control is indicated by*P< 0.05 and **P< 0.01.

## Slide 5
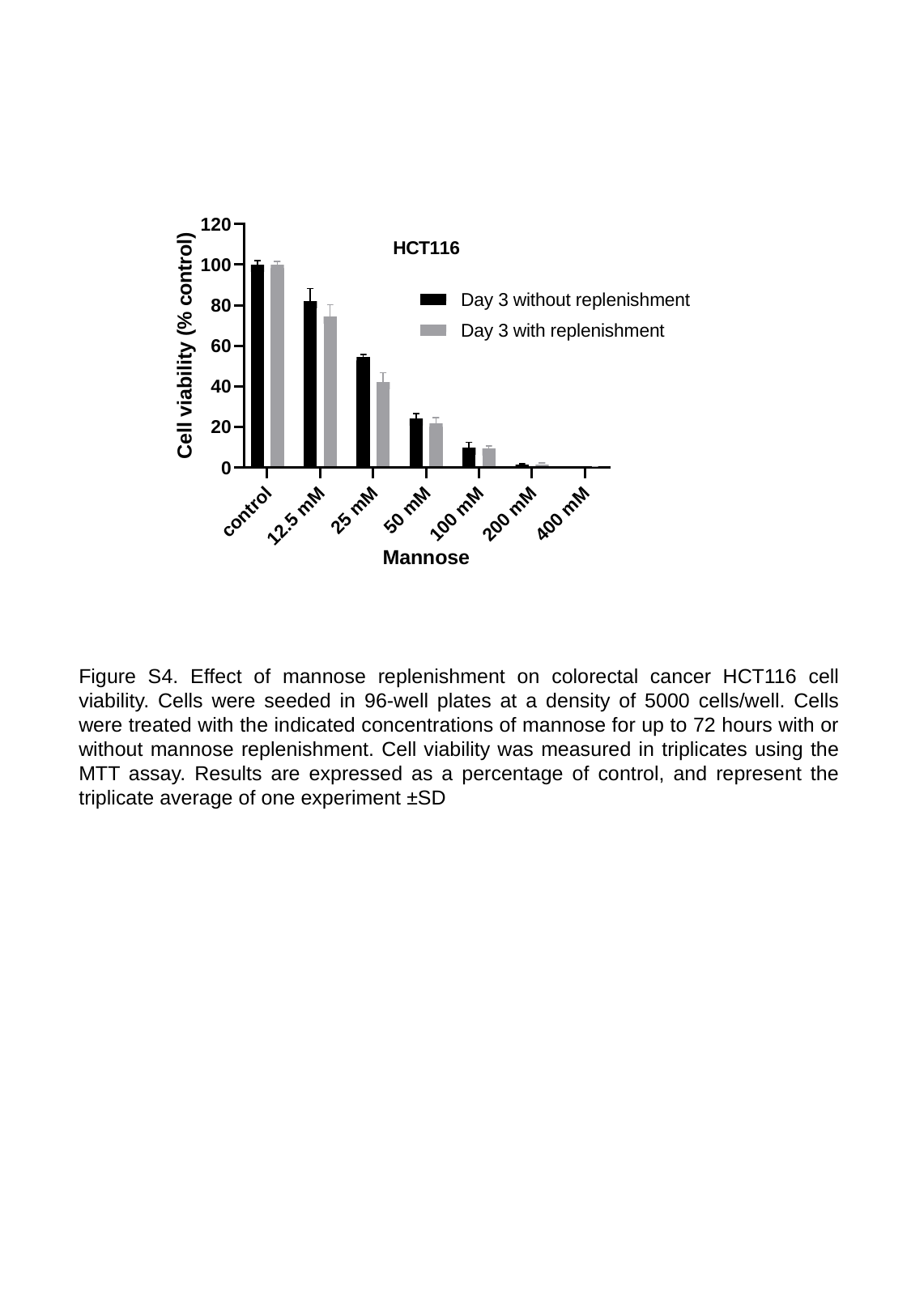

Figure S4. Effect of mannose replenishment on colorectal cancer HCT116 cell viability. Cells were seeded in 96-well plates at a density of 5000 cells/well. Cells were treated with the indicated concentrations of mannose for up to 72 hours with or without mannose replenishment. Cell viability was measured in triplicates using the MTT assay. Results are expressed as a percentage of control, and represent the triplicate average of one experiment ±SD

## Slide 6
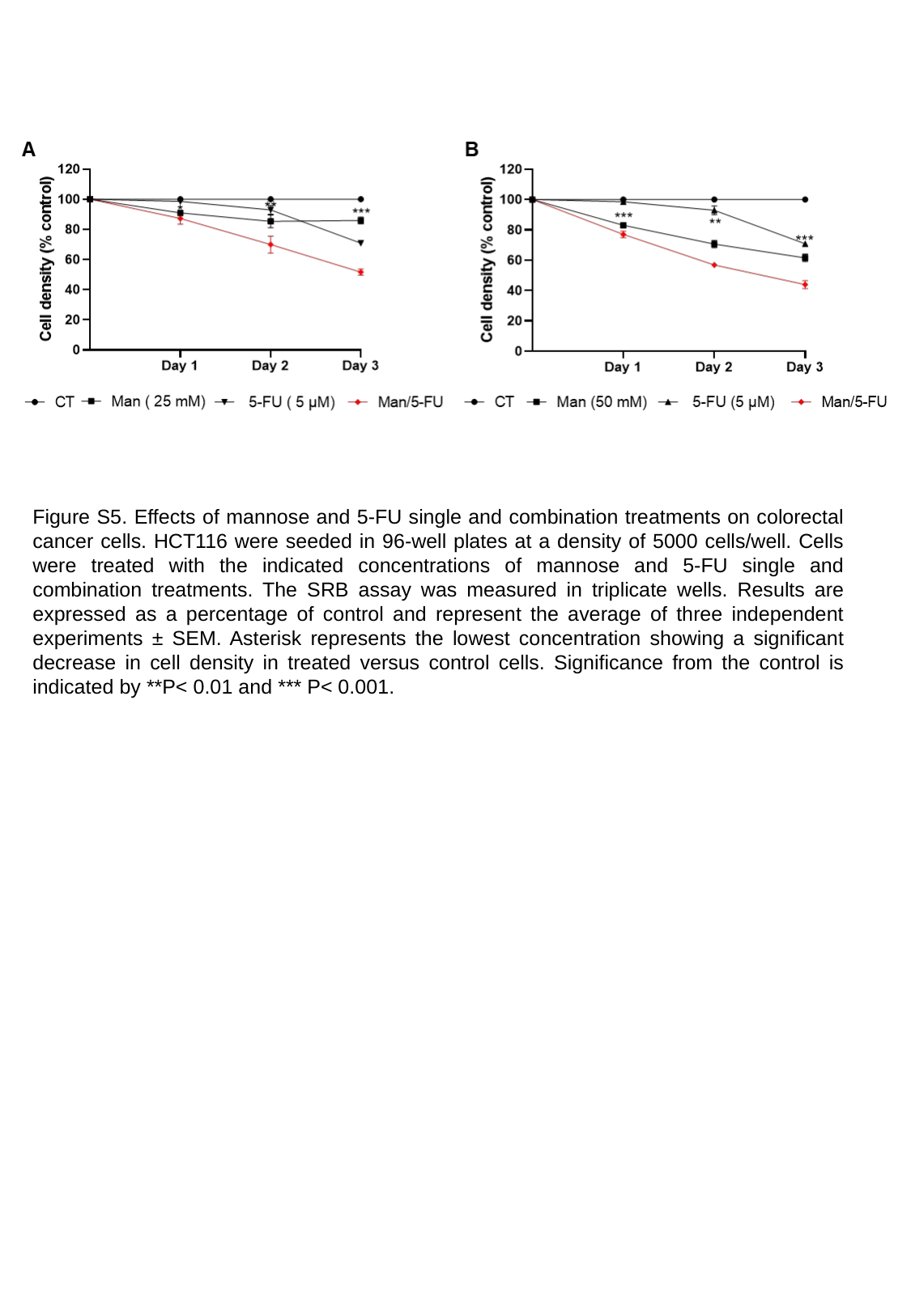

Figure S5. Effects of mannose and 5-FU single and combination treatments on colorectal cancer cells. HCT116 were seeded in 96-well plates at a density of 5000 cells/well. Cells were treated with the indicated concentrations of mannose and 5-FU single and combination treatments. The SRB assay was measured in triplicate wells. Results are expressed as a percentage of control and represent the average of three independent experiments ± SEM. Asterisk represents the lowest concentration showing a significant decrease in cell density in treated versus control cells. Significance from the control is indicated by **P< 0.01 and *** P< 0.001.

## Slide 7
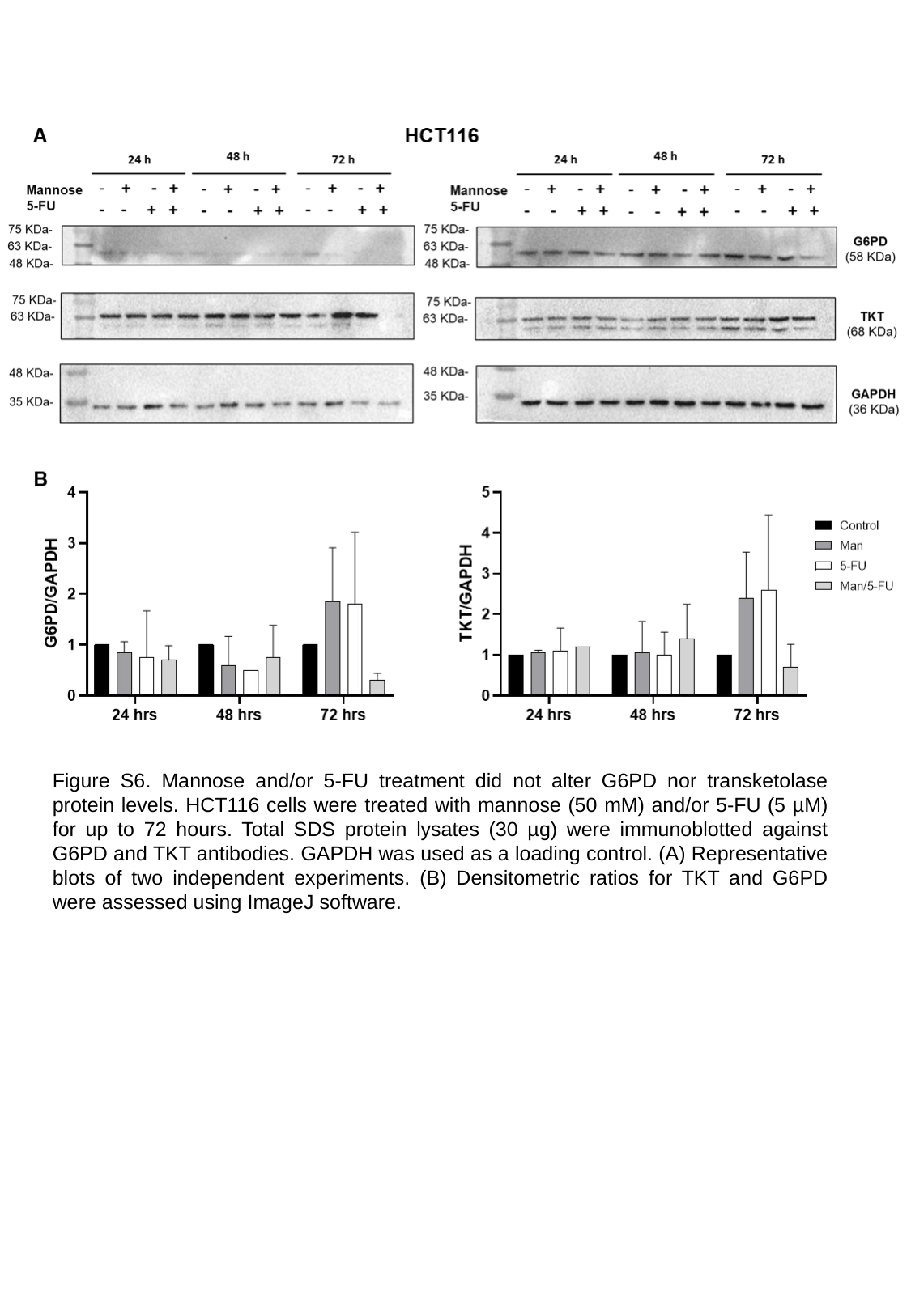

Figure S6. Mannose and/or 5-FU treatment did not alter G6PD nor transketolase protein levels. HCT116 cells were treated with mannose (50 mM) and/or 5-FU (5 µM) for up to 72 hours. Total SDS protein lysates (30 µg) were immunoblotted against G6PD and TKT antibodies. GAPDH was used as a loading control. (A) Representative blots of two independent experiments. (B) Densitometric ratios for TKT and G6PD were assessed using ImageJ software.

## Slide 8
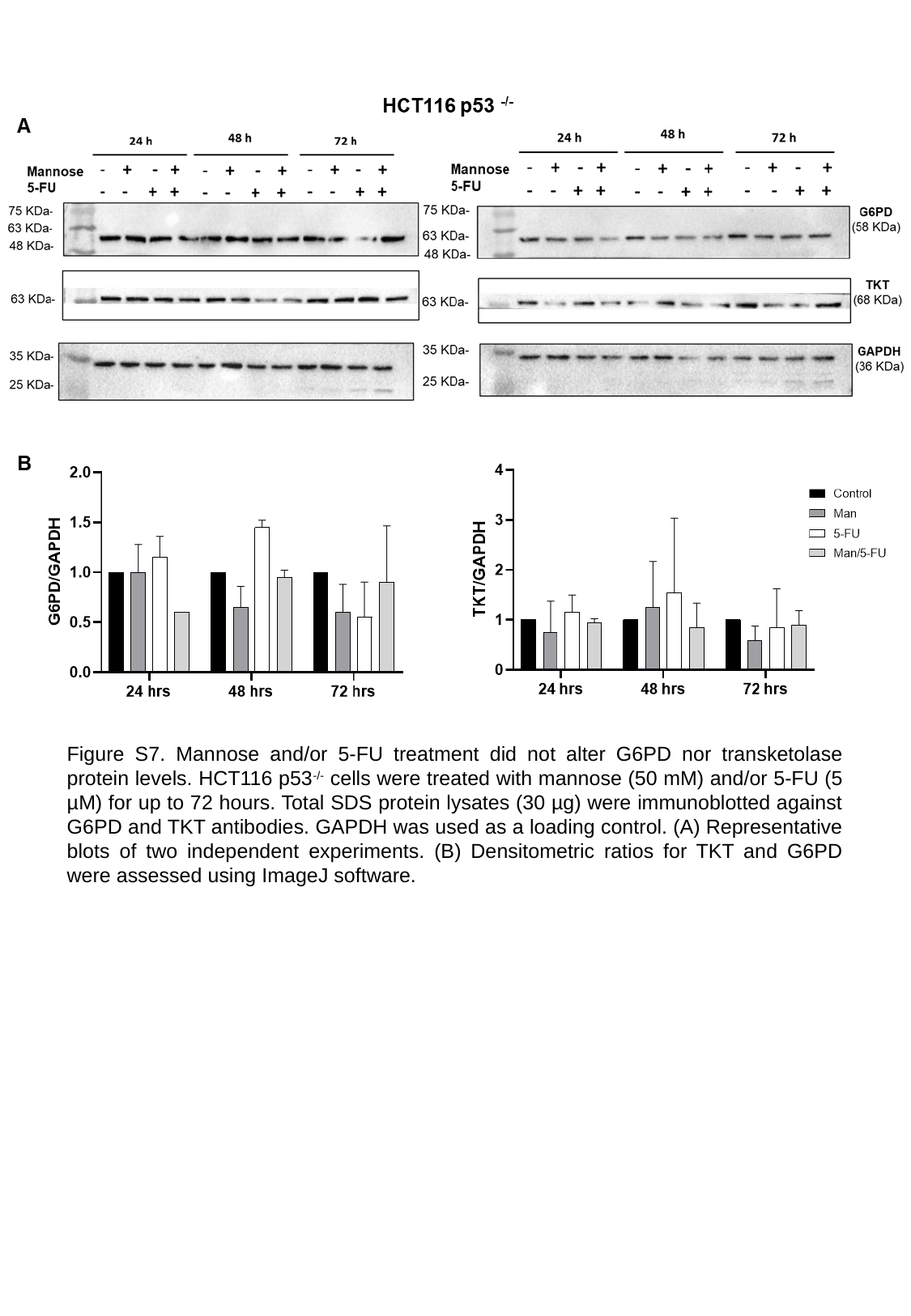

Figure S7. Mannose and/or 5-FU treatment did not alter G6PD nor transketolase protein levels. HCT116 p53-/- cells were treated with mannose (50 mM) and/or 5-FU (5 µM) for up to 72 hours. Total SDS protein lysates (30 µg) were immunoblotted against G6PD and TKT antibodies. GAPDH was used as a loading control. (A) Representative blots of two independent experiments. (B) Densitometric ratios for TKT and G6PD were assessed using ImageJ software.

## Slide 9
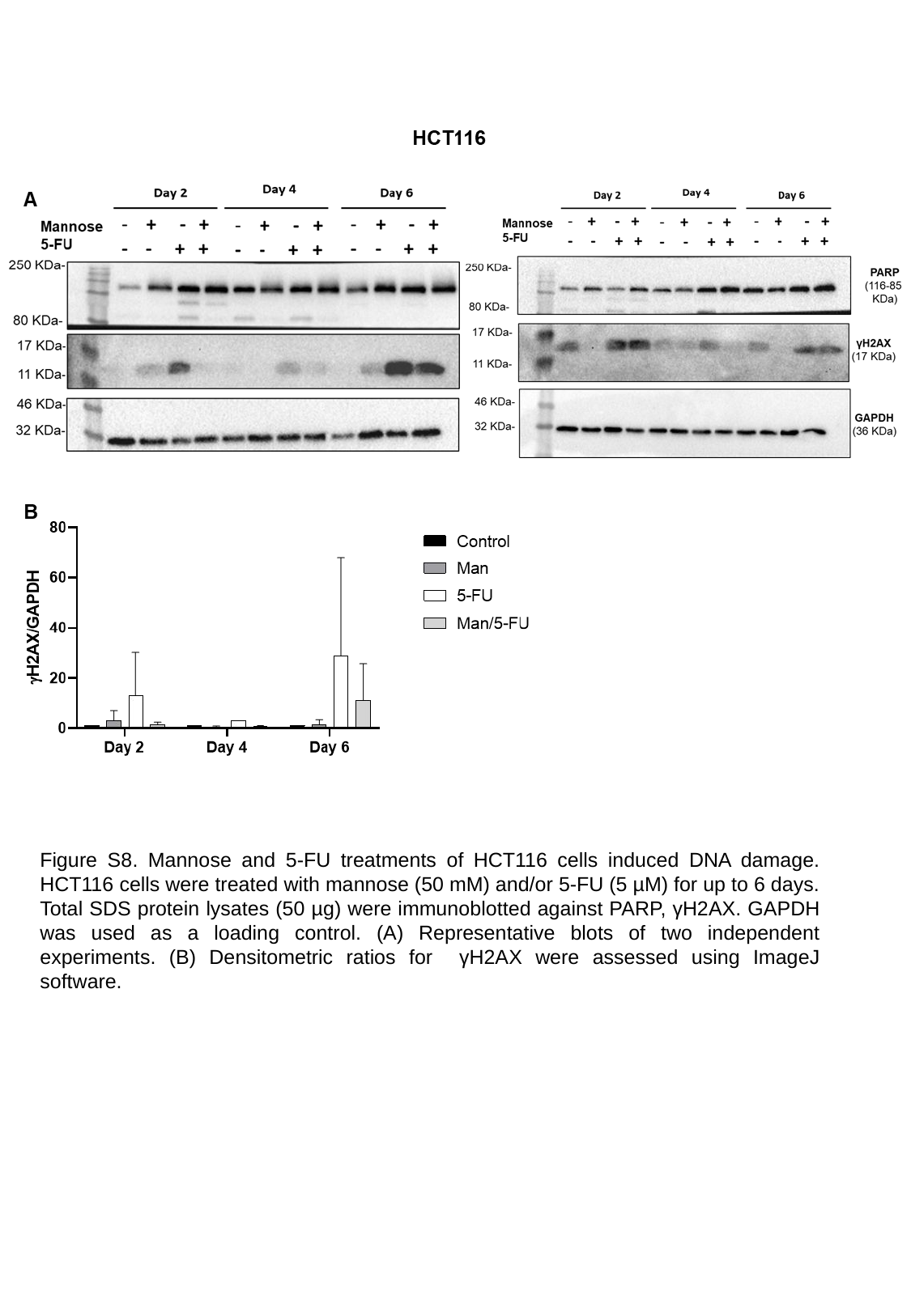

Figure S8. Mannose and 5-FU treatments of HCT116 cells induced DNA damage. HCT116 cells were treated with mannose (50 mM) and/or 5-FU (5 µM) for up to 6 days. Total SDS protein lysates (50 µg) were immunoblotted against PARP, γH2AX. GAPDH was used as a loading control. (A) Representative blots of two independent experiments. (B) Densitometric ratios for γH2AX were assessed using ImageJ software.

## Slide 10
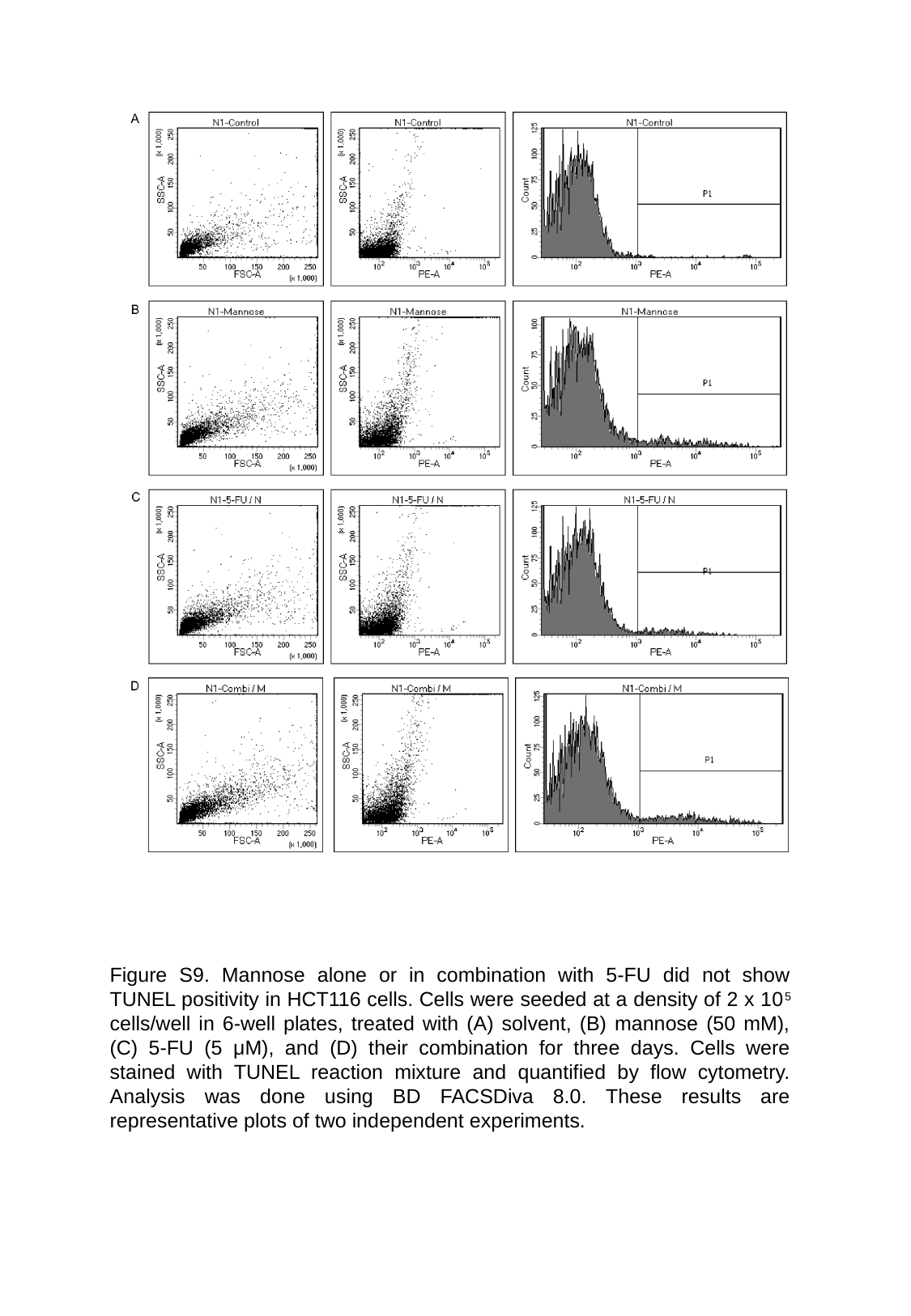

Figure S9. Mannose alone or in combination with 5-FU did not show TUNEL positivity in HCT116 cells. Cells were seeded at a density of 2 x 10^5^ cells/well in 6-well plates, treated with (A) solvent, (B) mannose (50 mM), (C) 5-FU (5 μM), and (D) their combination for three days. Cells were stained with TUNEL reaction mixture and quantified by flow cytometry. Analysis was done using BD FACSDiva 8.0. These results are representative plots of two independent experiments.
